# Compassion Fatigue as a Mediator Between Emotional Intelligence and Marital Anxiety Among Unmarried Mental Health Professionals Working in Family and Social Services

**DOI:** 10.3390/bs16060969

**Published:** 2026-06-11

**Authors:** Gamze Mukba, Serkan Oruç

**Affiliations:** Department of Psychological Counseling and Guidance, Van Yüzüncü Yıl University, 65080 Van, Turkey; serkanoruccc65@gmail.com

**Keywords:** emotional intelligence, compassion fatigue, marital anxiety, unmarried mental health professionals, mediation analysis

## Abstract

Professionals working in family and social services are frequently exposed to emotionally demanding interpersonal experiences, which may influence both their occupational well-being and their perceptions of close relationships. This study was conducted to examine the mediating role of compassion fatigue in the relationship between emotional intelligence and marital anxiety among unmarried mental health professionals in Türkiye. The sample consisted of 311 unmarried mental health workers, including psychologists, social workers, and psychological counselors employed in provincial directorates of the Ministry of Family and Social Services. Data were collected using the Trait Emotional Intelligence Questionnaire—Short Form (TEQue-SF), the Compassion Fatigue—Short Scale, and the Marital Anxiety Scale. Mediation analysis was conducted using PROCESS Macro Model 4. The findings revealed that emotional intelligence negatively predicted compassion fatigue. Emotional intelligence also negatively predicted marital anxiety, while compassion fatigue did not directly predict marital anxiety. Mediation analysis revealed that compassion fatigue played a significant moderate mediating role in the relationship between emotional intelligence and marital anxiety. These findings suggest that occupational emotional experiences may be indirectly associated with relationship-related concerns among unmarried mental health professionals. The results highlight the importance of considering both emotional intelligence and compassion fatigue in understanding marital anxiety and supporting the development of training, supervision, and psychoeducational interventions aimed at strengthening emotional regulation and professional well-being. Future research including both unmarried and married professionals, as well as longitudinal and mixed-method designs incorporating qualitative interviews, may further clarify these relationships and the mechanisms underlying them.

## 1. Introduction

Mental health professionals are routinely exposed to the psychological pain, trauma, and intense emotional experiences of others. While this exposure may contribute to positive outcomes for service users, it can also challenge the psychological resilience of practitioners and trainees and increase the risk of compassion fatigue and burnout (Malone, 2025; Paiva-Salisbury & Schwanz, 2022; Wachter et al., 2026). In professions requiring sustained empathic engagement, prolonged emotional labor may negatively affect subjective well-being, social relationships, and professional satisfaction, particularly when adequate training or supervision is lacking (Finzi-Dottan & Kormosh, 2018; Kounenou et al., 2023; Moudatsou et al., 2020; Wagaman et al., 2015).

Although mental health professionals are expected to maintain functional emotional boundaries, the gradual erosion of these boundaries may intensify internal emotional conflicts and psychological strain (Altun et al., 2020). Such strain has been linked to emotional exhaustion across caregiving roles, including social work, and may affect both occupational functioning and personal life satisfaction (Kinman & Grant, 2020; Kovbuz, 2025). Recent research has therefore emphasized the importance of mental health professionals’ capacity to regulate their own internal processes in addition to meeting professional responsibilities (Sharma & Jiwan, 2015).

For unmarried mental health professionals, limited social support and relational uncertainty may further intensify emotional strain. Emotional intelligence, defined as the ability to recognize and regulate emotions, plays a critical role in managing professional demands, maintaining interpersonal relationships, and coping with stress (Coronado-Maldonado & Benítez-Márquez, 2023; Issah, 2018; Salameh-Ayanian et al., 2025). Difficulties in balancing caregiving and self-care may increase emotion regulation problems and anxiety regarding long-term relationships or marriage (Newell & MacNeil, 2010).

Although studies involving married healthcare professionals have also reported findings related to marital anxiety and occupational burnout, marital anxiety appears to be more prominent among unmarried mental health professionals. In a study conducted by Chen et al. (2022), marital status was found to be associated with burnout among healthcare professionals; however, the influence of being married appeared to diminish when factors such as working conditions, leisure activities with spouses and family members, and available social support resources were taken into consideration.

The literature further indicates that continuous exposure to conflictual family relationships, divorce proceedings, and dysfunctional marital patterns in professional practice may lead unmarried professionals to develop more negative perceptions of the institution of marriage compared to their married counterparts. In a qualitative study conducted with married and unmarried social workers employed in family courts, Yüce and Yaman (2023) found that unmarried social workers reported greater marital anxiety, reservations about marriage, and a stronger tendency to postpone marriage than their married colleagues. Unmarried participants stated that frequent exposure to cases involving infidelity, family conflict, financial difficulties, and sexual problems diminished their trust in marriage and increased uncertainty and concerns regarding their future marital lives. In contrast, married participants reported that such cases had a more limited impact on their views of marriage and tended to evaluate these negative examples more cautiously, emphasizing that they did not represent all marriages (Yüce & Yaman, 2023).

Moore (2021) found that negative attitudes toward marriage among emerging counselors and therapists may be associated with a tendency to delay marriage. Moore (2021) also emphasized that individuals’ attitudes toward marriage can be shaped by family structure and prior experiences related to parental relationships. While Yüce and Yaman’s (2023) findings indicate that unmarried professionals may experience higher levels of marital anxiety, Moore’s (2021) doctoral dissertation demonstrated that attitudes toward marriage among mental health professionals are influenced by previous relationship experiences and family backgrounds. Taken together, these findings suggest that professionals without personal marital experience may experience greater uncertainty regarding the institution of marriage and may therefore be more vulnerable to marital anxiety than their married counterparts (Moore, 2021; Yüce & Yaman, 2023). Marriage anxiety refers to the experience of anxiety, negative emotional reactions, and maladaptive cognitions related to marriage, often accompanied by a tendency to avoid marriage and marriage-related thoughts (Çelik & Erkilet, 2019; Gul et al., 2025). Accordingly, this study examines the mediating role of compassion fatigue in the relationship between emotional intelligence and marital anxiety among unmarried mental health professionals working in family and social services in Türkiye.

### 1.1. Marriage Anxiety

Anxiety, defined as a state of tension arising from perceived uncertainty, may manifest in the marital context as fear regarding future relational expectations (Miceli & Castelfranchi, 2005; Şahin, 2019). Marriage anxiety in particular arises from perceived uncertainty or lack of control, influencing expectations and behaviors within intimate relationships. It may manifest as concern over future relational outcomes, fear of emotional investment, and worries about instability. Marital anxiety may also function as an avoidance-oriented construct among unmarried individuals when marriage is perceived as threatening or is associated with negative emotional experiences and adverse relational models observed in their social environment (Mesbah et al., 2025; Sönmez & Aktaş, 2023). Research shows that stressors in caregiving professions, such as high workload, limited income, and insufficient social support, create emotional strain that extends into personal life, contributing to conflict between work and family roles and ultimately increasing relational stress and anxiety (Amstad et al., 2011; Finzi-Dottan & Kormosh, 2018; Boyas & Wind, 2010).

In addition to occupational factors, broader social and relational experiences shape individuals’ perceptions of marriage. Exposure to divorce, family conflict, and relational instability may reinforce negative beliefs and maladaptive cognitive schemas (Tabkhi et al., 2025). These experiences, combined with the sustained emotional strain experienced in helping professions, may heighten marital anxiety, particularly among unmarried mental health professionals who are frequently exposed to clients’ relational difficulties (Bowman, 2025). In this context, professionals may be influenced by repeated exposure to their clients’ negative marital experiences and relationship narratives, which may shape their perceptions of marriage and contribute to greater uncertainty regarding their own future marriage relationships (Deacon et al., 1999). On the other hand, Moore (2021) emphasized that negative parental relationship patterns and family-of-origin experiences may adversely influence attitudes toward marriage among unmarried emerging therapists and counselors. Therefore, marriage anxiety can be conceptualized as a multidimensional construct shaped by the interaction of occupational stress, emotional processes, and social learning experiences in unmarried professionals.

### 1.2. Emotional Intelligence

Emotional intelligence, defined by Salovey and Mayer (1990) as the ability to recognize, evaluate, and regulate one’s own and others’ emotions and to use this information in thinking and problem solving, represents a core psychological capacity linked to emotional regulation, self-awareness, and interpersonal functioning (Avşar & Kaşıkçı, 2010; Salovey & Mayer, 1990). It supports adaptive responses to stress and is positively associated with job satisfaction, performance, organizational commitment, relationship quality, and effective management of emotional conflict (Hamulić et al., 2024; Uysal & Mammadov, 2020; Shafik, 2024). In caregiving professions such as psychology and social work, emotional intelligence is a critical competency due to continuous exposure to clients’ emotional difficulties. It facilitates therapeutic relationships, supports professional boundaries, and enhances intervention processes, promoting empathy and reflective practice and enabling adaptive, non-judgmental responses (Sizer & Parlak, 2021; Ackley, 2016; Herland, 2022; Matthews, 2022; Morrison, 2007).

Empirical evidence indicates that emotional intelligence functions as a protective factor against burnout by enhancing resilience and adaptive coping in mental health contexts (Chinchilla et al., 2024; Gill et al., 2011; Năstasă & Fărcaş, 2015). From a neurobiological perspective, processes such as emotional contagion, unconscious mimicry, and mirror neuron activation may lead individuals to internalize others’ emotions. Therefore, differentiating one’s own emotions from others is essential to maintain emotional clarity, prevent spillover, and sustain healthy interpersonal functioning (Weilenmann et al., 2018).

### 1.3. Compassion Fatigue

Compassion fatigue is characterized by emotional exhaustion, reduced empathic capacity, and diminished relational satisfaction resulting from prolonged exposure to suffering (Figley, 1995; Polat & Kaya, 2025; Tane et al., 2022). Figley (2002) conceptualized compassion fatigue as comprising secondary traumatization, burnout, and compassion satisfaction. In helping professionals, repeated engagement with traumatic material may lead to emotional depletion, heightened stress responses, and weakened empathic functioning (Coetzee & Klopper, 2010). Over time, particularly among caregiving professionals such as psychologists and social workers, compassion fatigue has been associated with physical, emotional, and psychological symptoms such as anxiety, depression, sleep disturbances, social withdrawal, diminished empathy, reduced responsiveness, and impaired relationships, as well as decreased satisfaction (Fontin et al., 2021; Kapoulitsas & Corcoran, 2015; Kırçı & Kızıler, 2021).

### 1.4. The Present Study

Professionals in helping fields such as psychologists, social workers, and psychological counselors are exposed to occupational stress and demanding cases requiring emotion recognition, regulation, and management, core components of emotional intelligence (Cadariu & Rad, 2025). Higher emotional intelligence has been associated with healthier empathic boundaries, more effective coping, and resistance to compassion fatigue, suggesting a protective role (Uysal & Mammadov, 2020). Conversely, lower emotional intelligence may limit self-awareness and self-care capacities, reduce stress management, and increase emotional exhaustion and relational anxiety (Shafik, 2024).

The spillover model provides a framework for understanding how occupational stress extends into personal and relational functioning. Mental health professionals working under sustained strain may carry emotional exhaustion into relationships, leading to heightened anxiety regarding long-term relationships or marriage (Finzi-Dottan & Kormosh, 2018). Within this framework, emotional intelligence may reduce the impact of occupational stress on relationships by supporting emotional awareness and regulation (Long et al., 2016). When these capacities are insufficient, stress may manifest as insecurity and anxiety related to emotional commitment.

Mental health professionals are routinely exposed to maladaptive family dynamics such as domestic violence, divorce, and emotional neglect (Nelson-Gardell & Harris, 2003). Repeated exposure may shape expectations regarding marriage and increase avoidance or distrust. Empirical evidence indicates that mental health professionals seek psychological support for anxiety, dyadic stress, and marital difficulties, while single professionals appear more vulnerable to occupational stress and depressive symptoms (Bike et al., 2009; Zheng et al., 2022). In this context, marital anxiety may emerge when emotional exhaustion and regulation difficulties coexist, particularly when emotional intelligence is insufficient (Kurniawan, 2019).

**Figure 1:**
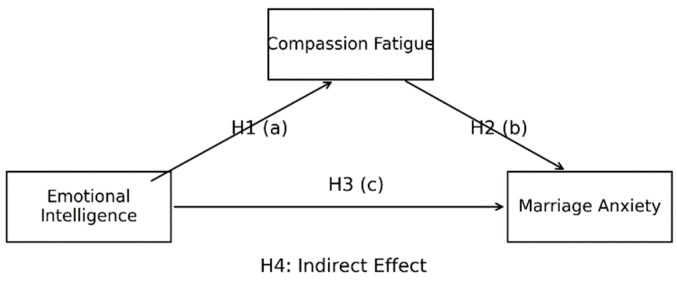
Direct and indirect paths.

Compassion fatigue may represent a central mechanism in this process. Professionals experiencing compassion fatigue may struggle to manage occupational stress and show reduced emotional intelligence, particularly in emotion regulation (Brillon et al., 2025). Mental health professionals working within Türkiye’s family and social service directorates address domestic violence, intrafamilial conflict, abuse, and marital issues to support relational well-being (Algan & Özmete, 2025; ASHB, 2023, 2024; Kutlu & Kutlu, 2024). Additionally, professionals working with such cases may experience work–family conflict (Ervüz & Türk, 2023). Therefore, this study examines the mediating role played by compassion fatigue in the relationship between emotional intelligence and marital anxiety among unmarried mental health professionals in Türkiye. The proposed direct and indirect paths, along with the study hypotheses, are presented in Figure 1.

**H1.**  *Emotional intelligence significantly predicts compassion fatigue.*

**H2.**  *Compassion fatigue significantly predicts marriage anxiety.*

**H3.**  *Emotional intelligence significantly predicts marriage anxiety.*

**H4.**  *Compassion fatigue mediates the relationship between emotional intelligence and marital anxiety.*

## 2. Method

### 2.1. Research Design

This study was designed to examine the mediating role played by compassion fatigue in the relationship between emotional intelligence and marriage anxiety among unmarried mental health professionals. A quantitative research approach was adopted. The proposed direct and indirect relationships among the study variables were tested using a regression-based path analysis framework. Specifically, mediation analysis was conducted using PROCESS Macro Model 4 for SPSS (Version 4; Hayes, 2022), which enables the estimation of direct and indirect effects through bootstrap procedures.

### 2.2. Participants

The participants consisted of unmarried mental health professionals (psychologists, psychological counselors, and social workers) working in Provincial Directorates of Family and Social Services affiliated with the Ministry of Family and Social Services across different cities in Türkiye. Their professional responsibilities include psychosocial assessment, counseling, case management, family support, and intervention services for individuals, couples, children, and families. As part of their professional duties, they frequently address domestic violence, intrafamilial conflict, child protection concerns, and marital difficulties in order to promote psychological well-being, strengthen family functioning, and support social welfare. Through these responsibilities, they provide preventive, protective, and supportive psychosocial services to vulnerable individuals and families within the community (ASHB, 2023, 2024; Gözen & Buz, 2020).

The sample was determined using the purposive sampling method. Purposive sampling is a non-probability sampling technique in which individuals are intentionally selected based on their relevance to the research purpose and their ability to meet specific inclusion criteria (Creswell, 2013). The inclusion criteria for participating in the study were as follows: (a) being unmarried, and (b) actively working in the field of mental health within provincial directorates of family and social services. Participants were described in terms of demographic variables such as age group, gender, and professional title. The frequency and percentage distributions related to participants’ gender, age groups, and professional titles are presented in Table 1 below.

The study sample consisted of 311 unmarried mental health professionals, of whom 54% were women and 46% were men.

### 2.3. Procedure

First, ethical approval for this study was obtained from the Social and Human Sciences Ethics Committee of Van Yüzüncü Yıl University (Decision No: 2025/16, dated 26 June 2025). Subsequently, data were collected between July and September 2025 from mental health professionals (psychologists, social workers, and psychological counselors) working in various provincial directorates of the Ministry of Family and Social Services across different cities, using both an online survey form and face-to-face interviews. Informed consent was obtained from all participants. A total of 311 mental health professionals who voluntarily participated in the study constituted the sample.

### 2.4. Data Collection Tools

#### 2.4.1. Compassion Fatigue—Short Scale

The Compassion Fatigue Short Scale was developed by Figley (1995) and adapted into Turkish by Yıldırım and Cavcav (2020). The scale consists of 13 items and two dimensions, namely occupational burnout and secondary traumatic stress, with a reported Cronbach’s alpha of 0.91. In the present study, the Compassion Fatigue Short Scale also demonstrated high internal consistency, with a Cronbach’s alpha coefficient of 0.921.

#### 2.4.2. Marriage Anxiety Scale

The Marriage Anxiety Scale was developed by Çelik and Erkilet (2019) as a 13-item, single-factor instrument with high internal consistency (Cronbach’s alpha = 0.93). In the present study, the Marriage Anxiety Scale demonstrated excellent internal consistency, with a Cronbach’s alpha coefficient of 0.928.

#### 2.4.3. The Trait Emotional Intelligence Questionnaire—Short Form (TEQue-SF)

The Trait Emotional Intelligence Questionnaire—Short Form was originally developed by Petrides and Furnham (2000, 2001) and adapted into Turkish by Deniz et al. (2013). The scale includes 20 items across four factors and demonstrated acceptable internal consistency and test–retest reliability. In the present study, the TEIQue-SF demonstrated acceptable internal consistency, with a Cronbach’s alpha coefficient of 0.780.

### 2.5. Data Analysis

SPSS 21.0 was used to assess the reliability of the measurement instruments through Cronbach’s alpha coefficients. Prior to hypothesis testing, the reliability of the measurement instruments was evaluated using Cronbach’s alpha coefficients. Pearson correlation analyses were conducted to examine the relationships among emotional intelligence, compassion fatigue, and marriage anxiety. The direct and indirect effects proposed in the research model were tested using PROCESS Macro Model 4. To examine the mediating role played by compassion fatigue in the relationship between emotional intelligence and marriage anxiety (H4), a bootstrap-based mediation analysis was conducted using the PROCESS macro for SPSS (Version 4; Hayes, 2022). Model 4 was specified, with emotional intelligence as the independent variable, marriage anxiety as the dependent variable, and compassion fatigue as the mediator. Indirect effects were evaluated using bias-corrected bootstrap confidence intervals based on 5000 resamples. The mediation effect was considered significant when the 95% confidence interval did not include zero (Creedon & Hayes, 2015; Hayes, 2022).

## 3. Findings

### 3.1. Descriptive Findings

The descriptive findings regarding the emotional intelligence, marital anxiety, and compassion fatigue levels experienced by mental health professionals are presented in Table 2 below.

Descriptive statistics and reliability coefficients for the emotional intelligence, marriage anxiety, and compassion fatigue scales are presented in Table 2. The Cronbach’s alpha coefficients ranged from 0.780 to 0.928, indicating acceptable-to-excellent internal consistency for all scales used in the study. The mean score for emotional intelligence was 95.20 (SD = 14.97), the mean score for marriage anxiety was 15.46 (SD = 8.17), and the mean score for compassion fatigue was 51.81 (SD = 26.28). Examination of skewness and kurtosis values indicated that all variables were within acceptable limits for normality. Specifically, skewness values ranged from −0.080 to 0.304 and kurtosis values ranged from −0.452 to −0.060. These findings suggest that the distributions of emotional intelligence, marriage anxiety, and compassion fatigue approximated normality.

### 3.2. Findings on the Relationship Between Emotional Intelligence, Compassion Fatigue, and Marital Anxiety

According to Table 3, the Pearson correlation analysis results regarding the relationships between emotional intelligence, compassion fatigue, and marriage anxiety are presented.

Table 3 shows that a negative and significant relationship was found between emotional intelligence and marriage anxiety (r = −0.17, *p* < 0.01). A negative and significant relationship was also identified between emotional intelligence and compassion fatigue (r = −0.52, *p* < 0.01). In addition, according to Table 3, a positive and significant relationship was found between marriage anxiety and compassion fatigue (r = 0.32, *p* < 0.05).

### 3.3. Findings Related to the Hypotheses

Based on the analyses conducted using PROCESS Macro for SPSS (Version 4; Hayes, 2022), the results of the direct and mediation path analyses are presented below. The results of the hypothesis tests for the direct paths are presented in Table 4.

H1 was supported. Emotional intelligence significantly and negatively predicted compassion fatigue among unmarried mental health professionals (β = −0.520, t = −10.718, *p* < 0.01, BootLLCI = −0.635, BootULCI = −0.402).

H2 was not supported. Compassion fatigue did not significantly predict marriage anxiety among unmarried mental health professionals (β = −0.009, t = −0.151, *p* = 0.880, BootLLCI = −0.133, BootULCI = 0.112).

H3 was supported. Emotional intelligence significantly and negatively predicted marriage anxiety among unmarried mental health professionals (β = −0.174, t = −3.106, *p* < 0.01, BootLLCI = −0.155, BootULCI = −0.035). After testing the direct paths, the mediating role played by compassion fatigue in the relationship between emotional intelligence and marriage anxiety was examined. The results of the mediation path analysis testing H4 are presented in Table 5.

**Figure 2:**
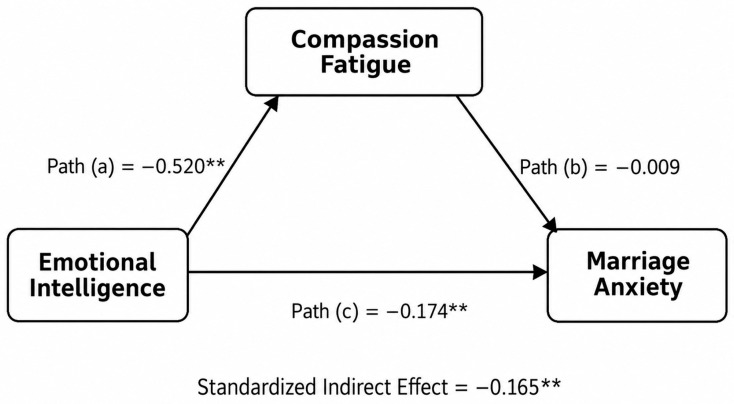
Mediation path analysis diagram. ** *p* < 0.01.

The mediation path analysis was statistically significant (F = 17.694, *p* < 0.001). The effect size of the mediation was moderate (η^2^ = 0.073, partial η^2^ = 0.075). Residual diagnostics indicated that the assumptions of normality (residual skewness = −0.0198, SE = 0.138; residual kurtosis = −0.240, SE = 0.276) and homogeneity of variance (Breusch–Pagan χ^2^ = 3.15, *p* > 0.05) were met. According to Table 5, H4 was supported. Compassion fatigue significantly mediated the relationship between emotional intelligence and marriage anxiety and explained 10.3% of the variance in marriage anxiety (R^2^ = 0.103). The standardized indirect effect was β = −0.165, with a standardized bootstrap confidence interval ranging from −0.244 to −0.093. Since the standardized bootstrap confidence interval did not include zero, the mediation effect was considered statistically significant. Given that η^2^ and partial η^2^ values were within the range of 0.06 to 0.14, compassion fatigue was found to have a moderate mediating role in the relationship between emotional intelligence and marriage anxiety. The mediation path analysis diagram is presented in Figure 2.

## 4. Discussion

Pearson correlation analyses revealed a negative relationship between emotional intelligence and marital anxiety, a negative relationship between emotional intelligence and compassion fatigue, and a positive relationship between compassion fatigue and marital anxiety among unmarried mental health professionals. The inverse association between emotional intelligence and marital anxiety suggests that unmarried professionals with higher emotional intelligence may more effectively integrate emotional regulation with personal expectations regarding marriage. Consistent with this finding, emotional intelligence has been linked to marital decision-making and relationship quality, including romantic satisfaction (Heidari & Kumar, 2021; Jardine et al., 2022; Kurniawan, 2019; Siavoshi et al., 2016).

The negative relationship between emotional intelligence and compassion fatigue indicates that unmarried mental health professionals with greater emotional awareness may develop healthier coping strategies without internalizing stress. Previous studies show that emotional awareness reduces secondary traumatic stress and enhances resilience and professional functioning (Grant & Kinman, 2014; Newell & MacNeil, 2010). Similar findings have been reported among mental health professionals (Kabunga et al., 2020; Yazıcı & Özdemir, 2023) and caregiving groups such as physicians and nurses (Kiran & Nuzhat-ul-Ain, 2024; Parker et al., 2024). Moreover, the interaction between emotional intelligence and compassion fatigue appears to influence emotion regulation, identification, and empathic functioning among helping professionals (Kaçan & Sakız, 2024; Noor et al., 2025). Previous research with psychologists and social workers shows that professional strain, limited social support, and a poor work–life balance are associated with emotional regulation difficulties and burnout (Ben-Zur & Michael, 2007; Çüm & Köroğlu, 2021; Stanley & Sebastine, 2023; Williams et al., 2020). In line with these findings, the negative association between emotional intelligence and compassion fatigue observed in the present study may reflect the buffering role of emotional awareness and self-regulation in managing occupational stress (Hamulić et al., 2024; Uysal & Mammadov, 2020). Although compassion fatigue represents the long-term emotional cost of empathic engagement (Figley, 2002), emotional intelligence may mitigate this cost and enhance resilience.

A positive relationship was found between compassion fatigue and marital anxiety among unmarried mental health professionals, suggesting that increases in compassion fatigue may heighten concerns about romantic relationships and marriage. In caregiving professions, this relationship may be explained by increased occupational stress, feelings of inadequacy, and depressive symptoms, which can interfere with relational functioning and reduce satisfaction (Monin et al., 2019). For unmarried professionals, such processes may shape expectations and anxieties about future relationships.

With the exception of H2, all hypotheses proposed in this study were supported. Findings related to H1 indicated that emotional intelligence significantly and negatively predicted compassion fatigue among unmarried mental health professionals. The ability to regulate, recognize, and express emotions adaptively may be inversely associated with professional fatigue in caregiving contexts. Supporting this interpretation, Beauvais et al. (2017) found that higher emotional intelligence and empathy toward positive emotions among nurses were associated with lower fatigue and burnout. Similarly, Ruiz Fernández et al. (2021) reported that emotional intelligence and perceived health explained compassion fatigue among healthcare professionals. When emotional intelligence is underdeveloped, emotional exhaustion is more likely. Villarosa-Hurlocker et al. (2019) also linked burnout among psychiatrists to emotional exhaustion, fatigue, and cynicism, which are related to emotional empathy.

Findings related to H2 indicated that compassion fatigue did not significantly predict marriage anxiety among unmarried mental health professionals. This finding may suggest that, although mental health professionals experience occupational fatigue and emotional burden in the course of their work, they may be able to cope effectively with concerns related to marriage through professional training, emotion regulation skills, and resilience resources. Baminiwatta et al. (2025) reported higher levels of resilience among married healthcare workers and highlighted resilience as a protective factor against burnout and its psychological consequences. Previous studies have similarly emphasized the protective role of adaptive coping strategies, social support, self-efficacy, and resilience in reducing the psychological impact of stressful life events and occupational challenges among healthcare professionals and individuals facing relationship-related difficulties (Gibbons et al., 2014; Negussie et al., 2023).

In contrast to the findings of this study, the “spillover of compassion fatigue into life domains” framework proposes that secondary traumatization and burnout may extend beyond occupational settings and negatively affect relational functioning and marital quality (Finzi-Dottan & Kormosh, 2016, 2018). Similarly, Negash and Sahin (2011) reported that compassion fatigue among marriage and family therapists may affect therapeutic processes and personal relationships. Etesaminia and Tarım (2025) also found a significant relationship between work–marriage conflict and compassion fatigue among healthcare professionals.

The results of H3 show that emotional intelligence had a negative and significant effect on marital anxiety among unmarried mental health professionals. Higher emotional intelligence may enable unmarried professionals to differentiate between emotional experiences in professional and personal domains, preventing the development of marital anxiety and supporting healthier relational expectations. Supporting this interpretation, emotional intelligence has been identified as a predictor of marital satisfaction (Eslami et al., 2014) and is associated with anxiety regulation, empathic communication, and relational trust (Deniz et al., 2013; Shafik, 2024). In contrast, lower emotional intelligence may increase vulnerability to mistrust and anxiety in relationships, whereas stronger emotion regulation skills may support relational stability across professional and personal contexts (Fookolaee et al., 2025; Ingram, 2013).

H4 was supported, indicating that compassion fatigue fully mediated the relationship between emotional intelligence and marital anxiety among unmarried mental health professionals. This suggests that the protective effect of emotional intelligence operates through reduced compassion fatigue. Lower emotional intelligence with higher compassion fatigue may increase marital anxiety, potentially through disruptions in cognitive schemas and relational trust (Buchanan et al., 2006; Pearlman & Mac Ian, 1995).

This mediating mechanism highlights a critical risk for unmarried mental health professionals: repeated exposure to clients’ marital conflicts, combined with difficulties in emotion regulation, may contribute to burnout and shape perceptions of romantic relationships and marriage. Supporting this perspective, research indicates that working with family conflict cases can influence social workers’ attitudes toward marriage and family life (Yüce & Yaman, 2023). Additionally, difficulties in managing occupational emotional burdens have been associated with increased anxiety, relational stress, and marital difficulties (Asfaw & Alene, 2023; Bike et al., 2009; Rose & Palattiyil, 2020). Exposure to clients’ emotional distress may intensify compassion fatigue, leading to emotional exhaustion and reduced intimacy in relationships (Venugopal et al., 2025). Taken together, these findings suggest that marital anxiety among unmarried mental health professionals is shaped by the combined effects of emotional intelligence and compassion fatigue. In this context, emotional regulation capacities appear central not only to professional functioning but also to how unmarried professionals construct expectations, trust, and anxiety regarding future relationships and marriage.

## 5. Limitations and Future Research

One limitation of this study is that the data were collected solely through quantitative methods. While this approach enabled the examination of relationships among emotional intelligence, compassion fatigue, and marital anxiety, it limited understanding of mental health professionals’ lived experiences. Another limitation is the study’s cross-sectional design, which precludes causal interpretations (Capili, 2021) of the observed relationships. Although significant associations and mediation pathways were identified, causal links among the study variables cannot be established. Future research may benefit from mixed-method designs, incorporating qualitative approaches such as interviews to provide a more comprehensive understanding about causal mechanisms of professional burnout, emotion regulation, and relational processes.

Another limitation concerns the sample, which consisted of unmarried mental health professionals working in provincial directorates of the Ministry of Family and Social Services across Türkiye. Cultural dynamics, including extended family structures and intergenerational patterns, may influence relational processes (Kagitcibasi et al., 2010; Özdemir Ocaklı & Yalçın, 2021) and experiences of compassion fatigue and marital anxiety, thereby limiting generalizability. Future research may examine this mediation across cultural contexts, explore how exposure to clients’ marital anxiety shapes professionals’ relational beliefs, and investigate the protective role of emotional intelligence. An additional limitation is that the sample consisted exclusively of unmarried mental health professionals. Therefore, the present study does not demonstrate whether the relationships among emotional intelligence, compassion fatigue, and marital anxiety differ according to marital status. Future research including both unmarried and married professionals may help clarify the potential role of marital status in these associations. Comparative and longitudinal studies across different relational statuses may further enhance understanding of how these relationships develop and change over time.

### Practice Implications

Work–family conflict may arise from imbalances between professional and marital domains, particularly when mental health professionals struggle to regulate and differentiate emotional demands across contexts (Chen et al., 2022). In this regard, emotional intelligence plays a key role in maintaining boundaries and managing occupational and relational stress. Based on the present findings, psychoeducational interventions may benefit from incorporating emotional intelligence, emotional awareness, and self-care components (McCade et al., 2021) to support mental health professionals in coping with these demands.

Such programs may also increase awareness of how exposure to clients’ relational conflicts influences personal beliefs and anxieties about relationships. Additionally, interventions including reflective and creative practices may help professionals avoid overidentification with caregiving roles and support personal growth (Huss & Sela-Amit, 2019). Overall, institutional support through resilience-focused, mindfulness-based, and supervision-oriented approaches may strengthen emotion regulation, reduce compassion fatigue, and promote a healthier work–life balance (Berger & Samuel, 2020; Chan & Wong, 2024; Choi, 2017; Kapoulitsas & Corcoran, 2015; Moudatsou et al., 2020). Accordingly, incorporating specific training on emotional intelligence, emotion regulation, and compassion fatigue into both academic education and workplace settings may further support professionals in coping with their own marital anxiety while maintaining healthy personal and professional boundaries. Furthermore, training and supervision opportunities may strengthen professionals’ competencies when working with complex family problems, conflicts between romantic partners, and family-related traumatic experiences.

## 6. Conclusions

In conclusion, this study contributes to the growing literature on the psychological well-being of unmarried mental health professionals working in family and social services by examining the relationships among emotional intelligence, compassion fatigue, and marital anxiety. The findings revealed that emotional intelligence negatively predicted both compassion fatigue and marital anxiety. Compassion fatigue did not directly predict marital anxiety. Mediation analysis revealed that compassion fatigue has a moderate mediating role in the relationship between emotional intelligence and marital anxiety. This finding suggests that occupational emotional experiences may be indirectly associated with relationship-related concerns. Taken together, these findings indicate that emotional intelligence and compassion fatigue are important factors in understanding marital anxiety among unmarried mental health professionals. The study further highlights the interconnected nature of occupational emotional experiences and personal relationship-related concerns, contributing to a better understanding of the psychological experiences of unmarried mental health professionals working with individuals, couples, and families.

## Figures and Tables

**Table 1:** Demographic characteristics of the participants.

Demographic Variable	Groups	N	%
Age Groups	23–35 years	188	60.5
	36 years and above	123	39.5
Gender	Female	168	54.0
	Male	143	46.0
Professional Title	Social Worker	83	26.7
	Psychologist	190	61.1
	Psychological Counselor	38	12.2

**Table 2:** Descriptive findings on emotional intelligence, compassion fatigue, and marriage anxiety among mental health practitioners.

Variable	α	N	Mean	SD	Skewness	Kurtosis
Emotional Intelligence	0.780	311	95.20	14.97	0.138	−0.452
Marriage Anxiety	0.928	311	15.46	8.17	−0.080	−0.297
Compassion Fatigue	0.921	311	51.81	26.28	0.304	−0.060

**Table 3:** Relationship between variables.

Variable	1	2	3
1 Emotional Intelligence	1	−0.174 **	−0.521 **
2 Marriage Anxiety		1	0.321 *
3 Compassion Fatigue			1

* *p* < 0.05; ** *p* < 0.01.

**Table 4:** Results of hypothesis testing for the direct paths.

Independent Variable	Path	Dependent Variable	H	β	T	*p*	BootLLCI ^1^	BootULCI ^1^
Emotional Intelligence	→	Compassion Fatigue	H1 (a)	−0.520	−10.718	*p* < 0.01	−0.635	−0.402
Compassion Fatigue	→	Marriage Anxiety	H2 (b)	−0.009	−0.151	0.880	−0.133	0.112
Emotional Intelligence	→	Marriage Anxiety	H3 (c)	−0.174	−3.106	*p* < 0.01	−0.155	−0.035

Note. β = standardized beta coefficient; ^1^ Bootstrap-based 95% confidence intervals.

**Table 5:** Results of the mediation path analysis.

H	IE	SIE	SE	SSE	R^2^	ΔR^2^	BootLLCI/BootULCI	Standardized BootLLCI/BootULCI
H4 (c′)	−0.090	−0.165	0.021	0.038	0.103	0.097	−0.134/−0.050	−0.244/−0.093

Note. IE = indirect effect; SIE = standardized indirect effect; SE = standard error; SSE = standardized standard error; BootLLCI and BootULCI = lower and upper limits of the bootstrap confidence interval; Standardized BootLLCI and Standardized BootULCI = lower and upper limits of the standardized bootstrap confidence interval; R^2^ = explained variance; ΔR^2^ = adjusted explained variance.

## Data Availability

Data available upon request.
